# Integration of Acute Malnutrition Treatment Into Integrated Community Case Management in Three Districts in Southern Mali: An Economic Evaluation

**DOI:** 10.9745/GHSP-D-23-00431

**Published:** 2024-06-27

**Authors:** Bernardette Cichon, Noemí López-Ejeda, Magloire Bunkembo Mampindu, Aliou Bagayoko, Mahamadou Samake, Pilar Charle Cuellar

**Affiliations:** a Action Against Hunger UK, London, United Kingdom.; b EPINUT Research Group, Unit of Physical Anthropology, Department of Biodiversity, Ecology and Evolution, Faculty of Biological Sciences, Complutense University of Madrid, Madrid, Spain.; c Action Against Hunger, Bamako, Mali.; d Nutrition Direction of the Ministry of Hygiene and Public Health, Bamako, Mali.; e Action Against Hunger Spain, Madrid, Spain.

## Abstract

**Background:**

Enabling community health workers (CHWs) to treat acute malnutrition improves treatment access and coverage. However, data on the cost and cost-effectiveness of this approach is limited. We aimed to cost the treatment at scale and determine the cost-effectiveness of different levels of supervision and technical support.

**Methods:**

This economic evaluation was part of a prospective nonrandomized community intervention study in 3 districts in Mali examining the impact of different levels of CHW and health center supervision and support on treatment outcomes for children with severe acute malnutrition. Treatment admission and outcome data were extracted from the records of 120 participating health centers and 169 CHW sites. Cost data were collected from accountancy records and through key informant interviews. Results were presented as cost per child treated and cured. Modeled scenario sensitivity analyses were conducted to determine how cost-efficiency and cost-effectiveness estimates change in an equal scale scenario and/or if the supervision had been done by government staff.

**Results:**

In the observed scenario, with an unequal number of children, the average cost per child treated was US$203.40 in Bafoulabé where a basic level of supervision and support was provided, US$279.90 in Kayes with a medium level of supervision, and US$253.9 in Kita with the highest level of supervision. Costs per child cured were US$303.90 in Bafoulabé, US$324.90 in Kayes, and US$311.80 in Kita, with overlapping uncertainty ranges.

**Conclusion:**

Additional supervision has the potential to be a cost-effective strategy if supervision costs are reduced without compromising the quality of supervision. Further research should aim to better adapt the supervision model and associated tools to the context and investigate where efficiencies can be made in its delivery.

## Abstract (precis)

Supportive supervision of health centers and community health workers providing treatment for acute malnutrition comes at significant costs but is needed to achieve good health outcomes. The most cost-effective way of delivering this treatment requires further research.

## Teaser

**Key Findings:**

- This study analyzed the costs associated with 3 levels (basic, medium, and high) of support to and supervision of acute malnutrition treatment at community health worker (CHW) sites and community health centers.
- The medium and high level of supervision significantly increased total program costs and cost per child treated compared to the basic level of supervision.
- However, as cure rates were significantly higher in the districts with medium or high levels of supervision, differences in cost per child cured were small with overlapping uncertainty ranges.
- Results suggest that, despite the higher costs, additional supervision is needed to achieve better treatment outcomes and that additional supervision has the potential to be a cost-effective strategy if efficiencies are made in its delivery.

**Key Implication:**

- Acute malnutrition treatment access, coverage, and health outcomes could be improved if adequate training, supervision, and technical support are in place and the best and most efficient way to deliver the additional supervision is carefully considered and is context specific to ensure cost-effectiveness and sustainability.

## Introduction

An estimated 45 million children worldwide are affected by wasting. It is linked to significant morbidity and mortality,^2^^,^^3^ and prevalence estimates of acute malnutrition have barely shifted over the last decade. The exact prevalence of acute malnutrition—a term that, in addition to children with a low weight-for-height z-score includes children with a mid-upper arm circumference (MUAC) of less than 125 mm and/or nutritional edema—is unknown but likely higher.

In the past, severe acute malnutrition (SAM), defined as a weight-for-height z-score less than −3 and a MUAC of less than 115 mm, required inpatient treatment solely at hospitals. This approach restricted the number of children that could receive treatment due to a limited number of hospital beds and required caregivers to spend a significant amount of time at the hospitals.^4^ In the early 2000s, the advent of ready-to-use therapeutic foods (RUTFs) and the introduction of community-based management of acute malnutrition enabled children to be treated at home as outpatients, requiring only weekly visits to health facilities. This approach has reduced treatment costs, increased coverage of treatment, and reduced mortality rates from SAM.^5^^–^^7^ Yet, it is estimated that less than 20% of children with acute malnutrition worldwide receive treatment as a result of unavailability of services, difficulty accessing services as a result of long distances to health centers, high opportunity costs to caregivers, and limited funds for acute malnutrition treatment services globally.^8^^,^^9^ Recently, there has been an increasing interest in approaches that reduce the cost, simplify treatment, and bring treatment closer to communities to increase the coverage of acute malnutrition treatment. One such approach is the integration of acute malnutrition treatment into the package of services provided by community health workers (CHWs) as part of the integrated community case management (iCCM) approach, which has shown promising results.^10^ The most recent revision of the World Health Organization guideline for the prevention and treatment of wasting and nutritional edema (acute malnutrition) stated that the identification and management of wasting can be carried out by CHWs if adequate training and supervision are provided.^11^ However, the certainty of evidence for this conditional recommendation was judged as very low,^11^ and more evidence is needed on the cost-effectiveness of this approach.^12^

Interest has increased in approaches that reduce the cost of acute malnutrition treatment, simplify treatment, and bring treatment closer to communities to increase coverage.

In Mali, one of the countries affected the worst by acute malnutrition, the treatment of SAM is already considered to be an important part of the package of activities to be provided by CHWs,^13^ and studies have shown the effectiveness of this approach both at small^14^ and large scale.^15^^,^^16^ An observational cohort study conducted in 7 health facilities and 19 CHW sites in Kita district, Kayes region in southwest Mali found that CHWs could effectively treat acute malnutrition in the community^14^ and that this approach was cost effective.^17^ Following the promising results of this pilot study, a second study was carried out from 2017 to 2018 at 120 health facilities and 169 CHW sites in 3 districts in the same region, where the 3 different districts received different levels of supervision as well as technical and financial support.^15^ The objective was to determine whether the results of the pilot study could be maintained at scale as well as what level of supervision support was needed to maintain a good quality of care and maximize cost-effectiveness. Results showed that district-level recovery rates ranged from 66.9% to 86.2%, where districts that received an increased amount of technical and financial support at both health centers and CHW sites and a greater level of supportive supervision of CHWs had better outcomes.^15^ We present the results of the economic evaluation linked to the aforementioned study.

## METHODS

### Study Context

This economic evaluation was linked to a prospective nonrandomized community intervention study in 3 districts in Mali, namely Bafoulabé, Kayes, and Kita in the region of Kayes, taking place between July 2017 and October 2018. The methods and outcomes of the parent study are described in more detail elsewhere.^15^

From 2016 to 2017, in Kayes, the prevalence of global acute malnutrition increased from 8.4% (95% confidence interval [CI]=6.3, 11.1) to 14.2% (95% CI=12.3, 16.4), and the prevalence of SAM increased from 0.9% (95% CI=0.5, 1.8) to 2.6% (95% CI=1.8, 3.6).^18^ A socioeconomic survey conducted as part of the study showed that there were no differences between the 3 districts in terms of the proportion of children aged younger than 5 years in the family, the proportion of children with a MUAC of less than 125 mm, and access to health care.^15^ However, in Bafoulabé, more households had access to clean water, a better food consumption score, and dietary diversity than households in the other 2 locations. Furthermore, the 3 districts differed in population size, density, and number of health facilities included (Table 1). In Bafoulabé, the educational level of CHWs was lower, but a greater proportion of CHWs had more than 2 years of experience there than in the other 2 locations (91.2% in Bafoulabé, 82.2% in Kayes, and 77.8% in Kita).^15^

**TABLE 1.** Characteristics of the Three Study Districts, Southwest Mali

	**Bafoulabé**	**Kayes**	**Kita**
Total population^a^	233,647	513,172	432,531
Size	19,808 km^2^	22,704 km^2^	35,626 km^2^
Population density^a^	11.8/km^2^	22.6/km^2^	12.1/km^2^
Participating health centers, no.	21	49	50
Participating CHW sites, no.	34	45	90

^a^Source: https://www.citypopulation.de.

### Intervention and Study Participants

In October 2017, the treatment of malnutrition was integrated into the iCCM model in 3 districts in Kayes, meaning that CHWs were able to detect and treat uncomplicated cases of SAM while health facilities also continued to provide treatment. All children aged 6–59 months with SAM defined as a MUAC less than 115 mm and/or a weight-for-height z-score (WHZ) less than −3 and/or edema admitted for treatment at 1 of the 120 health centers or 169 CHW sites were included in the study and received treatment according to the Malian nutrition protocol. Decentralizing treatment to CHW sites brings the treatment closer to the village level, thereby facilitating accessibility, as health centers are often difficult to reach. A CHW site typically is a room in the village given to the CHW by the community where they see patients and keep supplies. CHWs report to, are supervised by, and receive supplies, such as RUTF, from their affiliated health centers. They also refer complex cases beyond their capacity to the health centers. CHWs in the 3 districts received the same training, which consisted of a 3-week practical training and a 6-week supervised internship at the health facility, but different levels of supervision. Access to treatment in the project area before the intervention was low, with coverage surveys indicating a treatment coverage of 20.4% (12.9%–30.8%) in Bafoulabé, 28.7% (20.6%–38.6%) in Kayes, and 28.4% (19.9%–39.2%) in Kita.^19^ Low coverage was partly due to long distances to health facilities. In Mali, an estimated 42% of the population lives more than 5 km away from a health center.^20^ A year after the integration of acute malnutrition treatment into the package of care provided by CHWs, treatment coverage increased to 61.1% in Bafoulabé and increase to 57.1% in Kayes but did not change in Kita.^19^

### Supervision and Support

Intervention support and supervision differed across the 3 districts. In Bafoulabé, the basic level of supervision was implemented, which consisted of biannual supervision of the health centers and CHW sites by individuals from the national and regional Ministry of Health, quarterly supervision of the CHW sites by the district health management team of the Ministry of Health, and monthly supervision of the CHW sites by health center staff.

Intervention technical and financial support and biannual, quarterly, and monthly supervision visits differed across the 3 districts.

In Kita and Kayes, Action Against Hunger provided financial, logistic, and human resource support. This included financially supporting staff from the Ministry of Health and health centers to conduct biannual and quarterly supervision by covering per diems, transport, and accommodation costs. Furthermore, in these 2 districts, the quarterly supervision was expanded to also include supervision of health centers. Monthly supervision was also intensified in Kayes and Kita. In Kayes, the monthly supervision of CHW sites was done by 4 supervisors and 2 medical doctors employed by Action Against Hunger Mali, together with the health center technical directors using the supervision checklist from the iCCM policy in Mali.^13^ In Kita, CHW sites were subject to a particularly rigorous level of supervision, which, in addition to the same supervision received by CHW sites in Kayes, also included monthly nutrition-specific supervision visits by 6 supervisors and 3 medical doctors from Action Against Hunger using the checklist outlined in the Mali community-based management of acute malnutrition policy.^21^ The ratio of Action Against Hunger supervisors was 1 supervisor for 15 CHW sites in Kita and 1 for 11.2 CHW sites in Kayes. Action Against Hunger staff spent approximately half their time supervising CHW sites and half their time supporting the health centers. Table 2 provides an overview of the different levels of supervision in the 3 districts.

**TABLE 2.** Supervision Models Used at Health Centers and CHW Sites, Southwest Mali

	**Biannual Supervision**	**Quarterly Supervision**	**Monthly Supervision**	**Nutrition-Specific Supervision**
**Bafoulabé: Basic level of supervision**				
Target	Health centers and CHW sites	CHW sites	CHW sites	N/A
Approach	Approximately 8 days of supervision, including 3 days for problem sites, 1 day report writing/presentation and 2 for travel	Three sites visited during each supervision (12 sites over year)	Conducted with standard iCCM supervision checklist	
Team composition	3 people from national and regional Ministry of Health	3 people from district-level Ministry of Health	Team from health center and community health association	
**Kayes: Medium level of supervision**				
Target	Health centers and CHW sites	Health centers and CHW sites	CHW sites	N/A
Approach	Approximately 8 days of supervision, including 3 days for problem sites, 1 day report writing/presentation and 2 for travel	Approximately 15 sites visited during each supervision (60 over the year)	Conducted with standard iCCM supervision checklist	
Team composition	3 people from national and regional Ministry of Health	3 people from the district-level Ministry of Health, including chief doctor, and community health and social development focal points	Health center technical directors, a member of community health association, and 1 Action Against Hunger supervisor, who covers 10–12 CHW sites each (1 site per day)	
**Kita: High level of supervision**				
Target	Health centers and CHW sites	Health centers and CHW sites	CHW sites	CHW sites
Approach	Approximately 8 days of supervision, including 3 days for problem sites, 1 day report writing/presentation and 2 for travel	Team visits 1 health center and 1 CHW site per day—approximately 30 sites visited during each supervision (120 over the year)	Conducted with standard iCCM supervision checklist	Conducted with community-based management of acute malnutrition supervision checklist
Team composition	3 teams of 3 people including individuals from national and regional Ministry of Health	3 teams of 2–4 people, including district’s chief doctor, nutrition, community health, and social development focal points of the district level Ministry of Health	Health center technical directors, a member of community health association, and an Action Against Hunger supervisor. Each of the 6 Action Against Hunger supervisors covers 15 sites/month (1 site/day).	3 Action Against Hunger doctors supervised by district’s nutrition focal point conduct nutrition-specific supervisions, with each doctor covering 30 CHW sites with the aim to visit 2 sites/day and spend 1 week/month supporting the nutrition and community health focal points

Abbreviations: CHW, community health worker; iCCM, integrated community case management; N/A, not applicable.

### Design of the Economic Evaluation

Time horizons for the costing study are within the study period from July 2017 to October 2018. Training of CHWs took place between July and October 2017, and treatment from November 2017 to the end of October 2018. Financial and economic costs only for outpatient treatment incurred by project partners, health centers, and CHW sites were collected as part of this study. We did not include costs to families, as these were previously collected as part of the pilot study^14^ and were not expected to differ between the 3 supervision models.

### Cost Data Collection

Costs were estimated using an activity-based and ingredients approach. As an initial step, a focus group discussion with key project staff was conducted to determine project partners, timelines, project activities, and ingredients (Table 3).

**TABLE 3.** Overview of Cost Data Collected at CHW Sites and Health Centers, Southwest Mali

**Activity**	**Ingredients**
Trainings for CHWs and health center directors	Staff salaries (Action Against Hunger, government, health facility) CHW time Room rental Training materials Per diems, accommodation, and meals Transport costs
Supervision	Staff salaries (Action Against Hunger, government) Transport (car or motorbike usage, fuel) Per diem/ accommodation Supervisor meetings
Treatment (direct treatment costs)	Salaries (government and health center staff) CHW salaries Ready-to-use therapeutic foods, medicines Health center costs (space, upkeep, materials and running costs) CHW site costs (space, upkeep, materials and running costs)
Supply logistics	Salaries (Action Against Hunger, government and other partner staff). Transport to district level, from district to health center, and from health center to CHW site Storage at district level and health center
Coordination meetings (steering and technical committee)	Salaries (Action Against Hunger, government and partner staff) Room rental and food Transport (car motorbike usage and fuel) Per diem and accommodation
Action Against Hunger support costs	Support staff Office costs Accommodation and per diem

Abbreviation: CHW, community health worker.

Project proposals and documents were reviewed to understand which activities and ingredient costs would likely be found in the Action Against Hunger accountancy data and which additional costs needed to be determined through key informant interviews with project partners. The project was a collaboration between the Direction Nationale de la Santé (National Health Directorate) and the Direction Régionale de la Santé (Regional Health Directorate) of the Ministry of Health as well as the Institut National de Recherche en Santé Publique (National Public Health Research Institute) and Action Against Hunger, with essential supplies (e.g., RUTFs) provided by UNICEF. The CHW salaries were paid for by Save the Children with funding from the U.S. Agency for International Development. In addition, Population Services International played a role in equipping CHWs with essential tools, such as registers, support documents, and monthly reporting sheets. Finally, the Associations de Santé Communautaires (community health associations) manage the community health centers. Health center staff salaries are paid for either by the community health associations or the government.

Between September 24, 2018, and October 31, 2018, a total of 120 people were interviewed as part of this study. More specifically, at the national level, 20 people were interviewed from Action Against Hunger, Save the Children, Population Services International, the National Health Directorate, and the National Public Health Research Institute. At the regional level, 4 key informants from the Regional Health Directorate were interviewed. At the district level, 30 key informants (e.g., Action Against Hunger staff, district-level Ministry of Health staff, health center staff, and CHWs) were interviewed in Bafoulabé, 30 in Kayes, and 48 in Kita. Purposive sampling was used. Individuals were asked about costs, including supervision, staff, materials, supplies, transport, health center costs (including salaries, upkeep, equipment, and supplies), CHW site costs, and time spent on project activities, as well as time allocation to different project activities. To estimate the cost of the space used for the nutrition treatment program at the health centers and the CHW sites, respondents were asked what the monthly rent for such a space would be.

Data from a CHW time use study conducted as part of the main study were also used. This study was conducted among 140 CHWs (30 in Bafoulabé, 68 in Kita, and 42 in Kayes) to determine the total number of hours a CHW worked and the time spent on malnutrition treatment.^22^ CHWs were asked to note how much time they spent on iCCM activities for 1 month (September 1–30, 2018) using a self-administered questionnaire. Data showed that CHWs in Bafoulabé, Kayes, and Kita worked an average of 5 hours 10 minutes, 2 hours 40 minutes, and 2 hours 10 minutes per day, respectively. Of this, CHWs in Bafoulabé spent 13% of working hours on the treatment of SAM, CHWs in Kayes 12.3%, and CHWs in Kita 16.9%. Data from the time use study were triangulated with data from interviews with CHWs.

UNICEF provided costs per carton for RUTF supplies and associated transport costs. Total RUTF costs were estimated based on the cost per carton provided by UNICEF (US$45), the number of children admitted for treatment, and, due to the absence of RUTF consumption data, the assumption that 1 carton (150 sachets) per child was necessary for treatment.

Accountancy data from Action Against Hunger’s accounting system was reviewed at the end of the project. Any costs not directly linked to implementation, such as research costs, were excluded.

### Treatment Outcomes Data

Data on treatment outcomes were obtained from the main study.^15^ Outcomes included the number of children who recovered, defaulted, and died, as well as the number of children transferred to another health facility for inpatient care because they did not respond to treatment or developed another medical condition that prevented them from being treated as outpatients. Data were collected at CHW sites and health facilities. CHWs sent monthly activity reports to the health centers, which were reviewed by the health center technical directors and then sent to the district. At the district level, Action Against Hunger staff entered the data. Data from coverage surveys conducted at the start and the end of the study period, as well as data from socioeconomic surveys, were considered in the interpretation.^19^

### Data Management

Data from all sources (accountancy data, key informant interviews, time use study) were aggregated into a Microsoft Excel spreadsheet and adjusted.

Salary costs were obtained from Action Against Hunger accounting records for Action Against Hunger staff and from government salary scales for government and health center staff. Salary costs were then adjusted for time spent on the project, in the case of Action Against Hunger staff, or, in the case of health center staff, the proportion of time spent on malnutrition treatment. The proportion was determined during the key informant interviews or, in the case of CHWs, the time use study.

Building, facilities, and transport costs that were not supported by Action Against Hunger, such as CHW sites and health centers, were estimated based on interviews and adjusted according to the proportion of time spent on nutrition activities, as determined during key informant interviews and the CHW time use study conducted as part of the parent study.

Support costs from the Action Against Hunger accountancy were allocated to the 3 districts in several ways. First, costs that were independent of the number of staff or size of operation in the district were allocated as one-third to each district. Any support costs that were shared among multiple projects were allocated to each project based on a percentage of total projects. Costs that were expected to be dependent on the number of Action Against Hunger staff at the district level (e.g., support costs at the country office level) or number of CHWs (e.g., training) were allocated based on the percentage of personnel at Action Against Hunger district offices or CHWs in the districts, respectively.

Costs were adjusted for inflation and capital cost depreciation. Adjusting for inflation was done using the Consumer Price Index.^23^ Capital costs, defined as any item that can be used for more than 1 year and costs more than US$100,^24^ were adjusted to only reflect the costs of life of the item that was used by the program. Capital items, such as computers and cars, were amortized using standard tables and discounted at a rate of 3%. The lifespan of items was assigned as suggested by WHO-CHOICE.^25^ For office supplies not found in the WHO-CHOICE table, such as mobile phones and telephones, a life span of 3 years was assumed. As costs came from various sources and were expressed in different currencies, all costs were converted to US$ using average yearly exchange rates.^26^ All costs are expressed in 2018 US$.

### Data Analysis

Aggregated costs were organized into main cost centers according to activity and ingredients. Cost per child treated and cost per child cured were calculated by dividing the total cost per district by the number of children fully treated and cured, respectively. As the supervision of CHW sites was a key component of this study, we also presented CHW supervision and direct treatment costs (CHW time, CHW site costs, and supplies) separately (Supplemental Tables S1 and S2). However, it is important to note that the CHW sites do not function as an independent program and require a link to a functioning health center.

### Accounting for Uncertainty

Given the amount of assumptions regarding the inclusion and adjustment of costs, as well as estimations done when assigning economic costs, there is a lot of uncertainty in economic evaluations. In our analysis, we treated per diems and transport costs paid for by Action Against Hunger as fixed costs and RUTF, staff, support, and building and facilities costs as uncertain. Staff costs were treated as uncertain because of the difficulty in accurately estimating the amount of time spent on project activities in the absence of detailed time sheets. We assumed a 10% margin of error for costs collected during interviews,^27^ such as staff, support, building, and facilities costs. We assumed a −50% and +25% margin of error for RUTF based on data in the literature that showed a range of RUTF consumption between 70 and 193 sachets per patient,^15^^,^^28^^,^^29^ as well as the number of boxes sent to the study areas as provided by UNICEF, which is in line with the high estimate of 193 sachets per child.

We used an approach based on triangular fuzzy numbers and fuzzy arithmetic described by Myatt et al. to account for uncertainty and calculate 95% CI around cost-efficiency and cost-effectiveness estimates.^27^ Using triangular numbers to account for uncertainty is similar to a sampling based approach but is based on a triangular distribution instead of binomial or probability distributions.^27^ In this approach, triangular fuzzy numbers are expressed as a minimum, maximum, and most likely value, where the most likely value is the one estimated by the data collection in the base case, and the minimum and maximum were calculated as the base case +/− the assumed margin of error. For cure rates, the minimum and maximum were calculated as the proportion cured +/− 2 standard errors of the proportion.

Operations between fuzzy numbers (e.g., a combination of multiple cost categories and cost per child recovered as well as 95% CI) were calculated using a fuzzy arithmetic calculator.^30^

### Modeled Scenario Sensitivity Analyses

Three modeled scenario sensitivity analyses were conducted to determine how cost-efficiency and cost-effectiveness estimates change if the number of children treated and sites had been the same across locations and/or the supervision had been done by government staff. First, in the equal scale scenario sensitivity analysis, the number of sites and number of children treated per site from Kita was applied to the other 2 districts. RUTF costs and personnel time were adjusted to reflect the higher number of sites and children treated in Bafoulabé and Kayes. Cure rates from the main study were then applied to determine the cost per child cured. As there was a significant difference in the number of children treated at health facilities versus CHW sites,^15^ we recalculated the number of cured children separately for CHW sites and health facilities. In a second model, we reduced the Action Against Hunger supervision staff costs to one-third, a number that was deemed to be achievable by replacing supervision staff with government staff at the level of a nurse. The third model is a combination of the first 2 scenarios (i.e., equal scale and government staff costs).

### Ethical Approval

The study is registered under ISRCTN14990746. Ethical approval was obtained from the Ethics board in Mali (No. 17/2017/CE-INRSP).

## RESULTS

### Cohort Treatment Outcomes

During the study duration, a total of 862, 1,930, and 3,320 children were treated in Bafoulabé, Kayes, and Kita, respectively. The treatment outcomes are described in detail by Charle et al.^15^ Nevertheless, the main results are reproduced here to facilitate an understanding of the economic evaluation results. The number of children by district and average number of children treated by health facility and CHW is shown in Table 4, and the treatment outcomes are shown in Table 5.

**TABLE 4.** Number of Children Treated for Acute Malnutrition at Health Centers and by CHWs, Southwest Mali

	**Bafoulabé**	**Kayes**	**Kita**
Children included in study sample, no.	862	1,930	3,320
Children treated at health centers, no. (%)	549 (63.7)	1,580 (81.9)	2,793 (84.1)
Children treated by CHWs, no. (%)	313 (36.3)	350 (18.1)	527 (15.9)
Children treated per health center, average	29	32	56
Children treated per CHW, average	9	8	6

Abbreviation: CHW, community health worker.

**TABLE 5.** Outcomes for Children Treated for Acute Malnutrition, Southwest Mali

	**Bafoulabé**	**Kayes**	**Kita**
**All children**			
Treated, no.	862	1,930	3,320
Cured, no.	577	1,663	2,704
Cured, % (95% CI)	66.9 (63.8, 70.1)	86.2 (84.6, 87.7)	81.4 (80.1, 82.8)
Defaulted, % (95% CI)	15.3 (12.9, 17.7)	1.8 (1.1, 2.3)	6.4 (5.6, 7.2)
Transferred, % (95% CI)	16.9 (14.4, 19.4)	11.9 (10.5, 13.4)	11.7 (10.7, 12.8)
Died, % (95% CI)	0.8 (0.2, 1.4)	0.1 (0.04, 0.3)	0.4 (0.2, 0.6)
**Community health worker sites**			
Treated, no.	313	350	527
Cured, no.	227	303	412
Cured, % (95% CI)	72.5 (67.6, 77.5)	86.9 (83.3, 90.4)	78.2 (74.6, 81.7)
Defaulted, % (95% CI)	6.4 (3.7, 9.1)	5.7 (3.3, 8.2)	6.6 (4.5, 8.8)
Transferred, % (95% CI)	21.1 (16.5, 25.6)	7.4 (4.7, 10.2)	14.8 (11.8, 17.8)
Died, % (95% CI)	0	0	0.4 (0.2, 0.9)
**Health centers**			
Treated, no.	549	1,580	2,793
Cured, no.	350	1,360	2,295
Cured, % (95% CI)	63.8 (59.7, 67.8)	86.1 (84.4, 87.8)	82.2 (80.7, 83.6)
Defaulted, % (95% CI)	20.4 (17.0, 23.7)	0.8 (0.4, 1.3)	6.3 (5.4, 7.2)
Transferred, % (95% CI)	14.6 (11.6, 17.5)	12.9 (11.3, 14.6)	11.2 (10.0, 12.3)
Died, % (95% CI)	1.3 (0.03, 2.2)	0.1 (0.05, 0.3)	0.4 (0.2, 0.6)

Abbreviation: CI, confidence interval.

### Total Intervention Costs

In the observed scenario, the total cost of the intervention in Bafoulabé was US$175373.8 (95% CI=US$159839.9, US$191948.4); in Kayes, US$540270.6 (95% CI=US$476549, US$588147.6); and in Kita, US$843084.6 (95% CI=US$734885.4, US$912231.8), with treatment and supervision being the most expensive activities (Table 6). In terms of ingredients, personnel costs make up the highest proportion of total costs in the 3 intervention areas. Treatment supplies (RUTFs and medicines) make up 23.5%, 17.2%, and 18.4% of total costs in Bafoulabé, Kayes, and Kita, respectively. The costs of CHW salaries make up only 2.1% of total project costs in Bafoulabé, 0.7% in Kayes, and 1.7 % in Kita (Table 6 and Figure). Direct treatment costs accounted for 51.1% in Bafoulabé, 34.0% in Kayes, and 38.7% in Kita (Table 6).

**TABLE 6.** Project Costs by Activity for Each Study Location in the Base Case, Southwest Mali

**Activities and Ingredients**	**Bafoulabé**	**Kayes**	**Kita**
**Trainings subtotal, US$ (% of total)**	**26791 (15.3)**	**34199 (6.3)**	**62092 (7.4)**
CHW sites			
Cost of training staff	3903.5 (2.2)	5171.1 (1)	10908.4 (1.3)
Cost of room rental, materials, and supplies	1485 (0.8)	1869 (0.3)	3576.0 (0.4)
Per diem and transport	19959.9 (11.4)	23930.8 (4.4)	44242.0 (5.3)
Training of health center directors	1442.3 (0.9)	3228.0 (0.6)	3365.4 (0.4)
**Supervision subtotal, US$ (% of total)**	**27819 (15.9)**	**174551.1 (32.3)**	**232047.8 (27.5)**
Action Against Hunger staff time^a^	12788.8 (7.3)	138635.7 (25.6)	154373.6 (18.3)
Directorate of Health and community health association staff time^a^	3304.4 (1.9)	2434.0 (0.5)	8309.5 (1.0)
Health center staff time^b^	8704.2 (5.0)	9451.6 (1.7)	23146.7 (2.8)
Transport, communication, supplies	3021.6 (1.7)	10603.6 (2.0)	22705.3 (2.7)
Per diem and accommodation	0.0	8096.7 (1.5)	18183.1 (2.1)
Supervisor meetings	0.0	5329.5 (1.0)	5329.5 (0.6)
**Direct treatment costs subtotal, US$ (% of total)**	**89658.4 (51.1)**	**183721.8 (34.0)**	**323047.8 (38.7)**
Health center staff salaries	29508.5 (16.8)	57377.7 (10.6)	97173.1 (11.5)
CHW salaries	3705.6 (2.1)	3867.0 (0.7)	14638.0 (1.7)
RUTFs and medicine	41244.0 (23.5)	92692.0 (17.2)	154657.0 (18.4)
Health center costs (space and upkeep)	2090.7 (1.2)	5391.80 (1.0)	6025.82 (0.7)
CHW site costs (space and upkeep)	572.2 (0.3)	966.8 (0.2)	3187.9 (0.4)
Active case finding	0.0 (0)	1465.6 (0.3)	2845.0 (0.3)
Materials and supplies	12537.4(7.2)	21960.9 (4.1)	47752.7 (5.7)
**Supply logistics subtotal, US$ (% of total)**	**14315 (8.2)**	**14932 (2.8)**	**22257 (2.6)**
Personnel	818.3 (0.5)	618.0 (0.1)	1422.2 (0.2)
Transport (RUTF)	8021.2 (4.6)	9685.9 (1.8)	14756.4 (1.8)
Storage of supplies	5475.6 (3.1)	4628.5 (0.9)	6078.2 (0.7)
**Coordination meetings of the steering and technical committee, US$ (% of total)**	**8467 (4.8)**	**8426 (1.6)**	**8500 (1.0)**
Personnel	5744.2 (3.2)	5677.2 (1.1)	5744.2 (0.7)
Room rental, per diem, accommodation	1719.9 (1.0)	1719.9 (0.3)	1719.9 (0.2)
Transport	1003.1(0.6)	1028.7 (0.2)	1035.7 (0.1)
**Action Against Hunger support costs, US$ (% of total)**	**8324 (4.8)**	**124440 (23.0)**	**191910 (22.8)**
Personnel	8317.7 (4.7)	107755.0 (19.9)	164433.2 (19.5)
Per diem and accommodation	0.0 (0)	1535.0 (0.3)	1445.0 (0.2)
Transport	0.0 (0)	7002.8 (1.3)	13357.3 (1.6)
Office costs	5.8 (<0.1)	8147.4 (1.5)	12.674.2 (1.5)
**Total, US$ (% of total)**	**175373.8 (100)**	**540270.6 (100)**	**843084.6 (100)**
Cost to Action Against Hunger	50610 (28.9)	330506.63 (61.2)	458443.4 (54.3)
Cost to other international NGOs and UN agencies^c^	63196.4 (36.0)	119893.6 (22.2)	216855. (25.7)
Cost to the government, community health association, and community	61567.5 (35.1)	89870.4 (16.6)	167786.7 (19.9)

Abbreviations: CHW, community health worker; NGO, nongovernmental organization; RUTF, ready-to-use therapeutic food; UN, United Nations.

^a^Includes supervision to CHW sites and support/supervision of health centers.

^b^Supervision of CHW sites only.

^c^These include Save the Children, Population Services International, and UNICEF.

**FIGURE:**
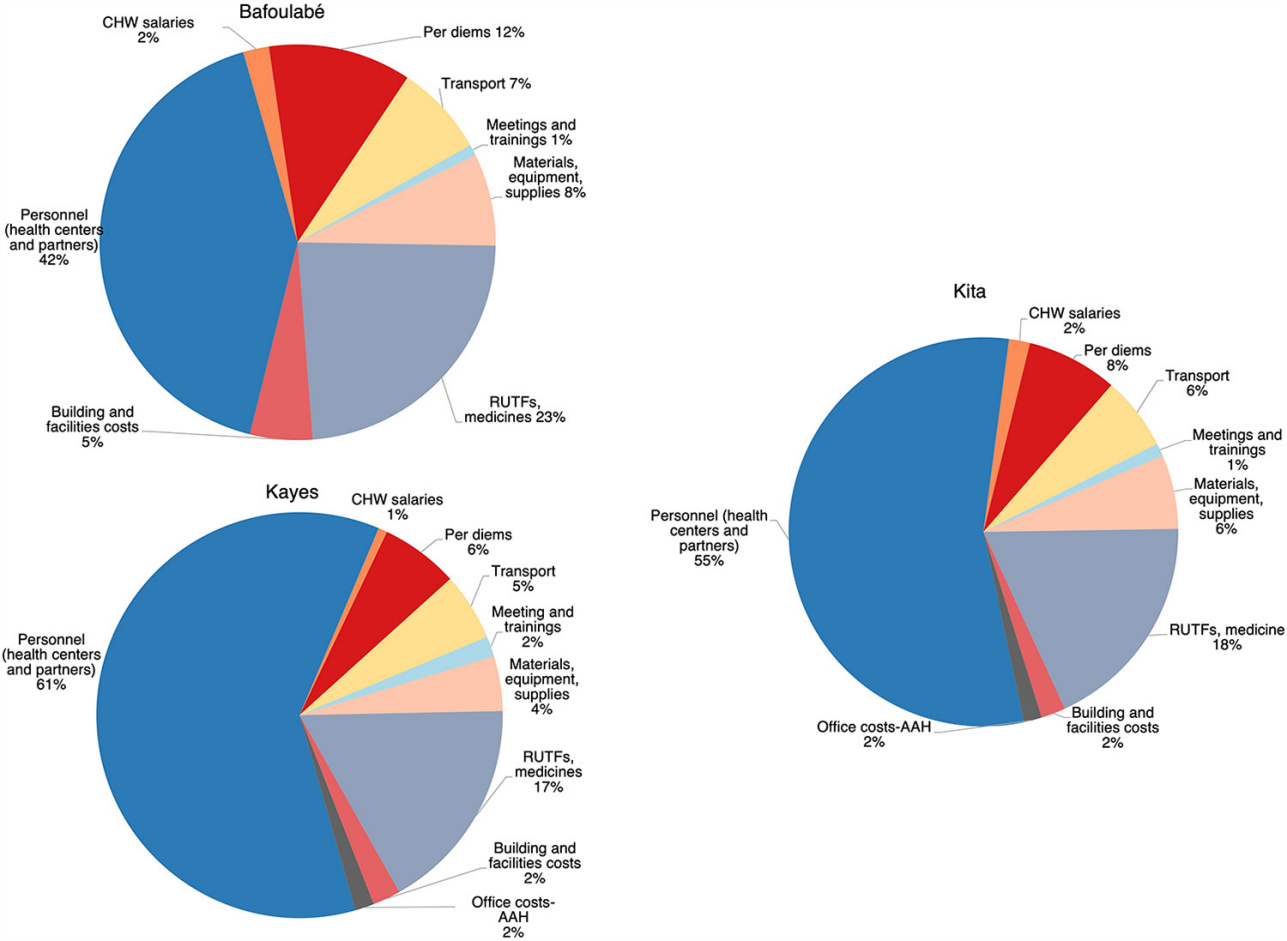
Costs Associated With Integrating Treatment of Acute Malnutrition Into Integrated Community Case Management, by Ingredient, in Three Districts, Southwest Mali Abbreviations: AAH, Action Against Hunger; CHW, community health worker; RUTF, ready-to-use therapeutic food.

### Supervision Costs

Supervision costs amounted to US$27819 (15.9%), US$174551.1 (32.3%), and US$232047.8 (27.7%) in Bafoulabé, Kayes, and Kita, respectively (Table 6). Of the total supervision costs, US$10939.4 (41.6%), US$60919.2 (34.6%), and US$123897.5 (52.2%) were spent on supervision of CHWs in Bafoulabé, Kayes, and Kita; the rest was supervision to the health centers. This equates to a monthly supervision cost per CHW of US$27 in Bafoulabé, US$113 in Kayes, and US$115 in Kita. A breakdown of CHW supervision costs, as well as supervision costs per child treated and recovered at CHW sites, is presented in Supplemental Table S1.

### Cost per Child Treated and Recovered

In the base case, the average cost per child treated was US$203.4 (95% CI=US$185.4, US$222.7) in Bafoulabé; US$279.9 (95% CI=US$246.9, US$304.7) in Kayes; and US$253.9 (US$221.4, US$274.8) in Kita. A total of US$48 (95% CI=US$28, US$57) of this were RUTF and medicine costs. There was no difference between the 3 locations as the estimates of RUTF data were based on the literature rather than consumption during the study. Lack of available data on RUTF consumption led to a large amount of uncertainty in RUTF cost estimates; therefore, the cost per child treated and cured are presented both with and without RUTF costs (Table 7). In the base case, the cost per child treated in Bafoulabé was notably lower than in the other 2 districts, the difference in cost per child recovered was less pronounced, and uncertainty ranges overlapped (Table 7). Direct treatment costs per child treated and cured disaggregated by treatment site are shown in Supplemental Table S2.

**TABLE 7.** Cost per Child Treated and Cured in the Observed Base Case Analysis, Southwest Mali

	**Bafoulabé, US$ (95% CI)**	**Kayes, US$ (95% CI)**	**Kita, US$ (95% CI)**
Total cost	175373.8 (159839.9, 191948.4)	540270.6 (476549, 588147.6)	843084.6 (734885.4, 912231.8)
Total cost per child treated	203.4 (185.4, 222.7)	279.9 (246.9, 304.7)	253.9 (221.4, 274.8)
Excluding RUTF costs	155.6 (140.9, 165.2)	231.9 (218.2, 248.0)	207.4 (193.5, 219.8)
Total cost per child recovered	303.9 (267.6, 346.4)	324.9 (282.9, 358.9)	311.8 (268.6, 342.0)
Excluding RUTF costs	232.5 (203.3, 256.9)	269.1 (249.9, 292.1)	254.6 (234.7, 273.5)

Abbreviations: CI, confidence interval; RUTF, ready-to-use therapeutic food.

### Modeled Scenarios

The equal scale modeled scenario sensitivity analysis showed that, where all costs not related to the additional supervision were equal, such as the number of health centers, the cost per child treated and cured in Bafoulabé was significantly lower than in the other 2 areas. In contrast, where Action Against Hunger supervision staff costs are replaced by government staff costs, the additional supervision and support provided in Kita and Kayes becomes a cost-effective strategy (Supplemental Table S3).

## DISCUSSION

In this study, we have assessed the cost of the treatment of acute malnutrition in 3 districts in southern Mali and aimed to determine the impact of additional supervision and technical support at CHW sites and health centers on the cost per child treated and cost per child cured.

Overall, our treatment cost estimates, ranging from US$203 to US$280, were comparable to estimates in previous studies, ranging from US$135–US$802 in Zambia,^31^ Malawi,^6^ Ethiopia,^7^ Niger,^32^^,^^33^ Ghana,^34^ Bangladesh,^5^ Mali,^17^ and Pakistan.^35^ An earlier smaller study in Kita, Mali, covering some of the same sites as this study, found a similar cost per child treated and cured of US$244 and US$259, respectively.^17^ Similar to other studies involving international nongovernmental organizations (NGOs),^5^^,^^17^^,^^31^^,^^35^ we found that institutional support, coordination, and supervision costs together accounted for a large proportion of overall costs, in this case, more than 50% in Kayes and Kita. In Bafoulabé, without the additional technical and supervision support, these costs were lower at 25.5%, and the proportion of direct treatment costs was higher (51.1% Bafoulabé, compared to 34% in Kayes and 39% in Kita). In terms of ingredients, the largest proportion of costs were personnel costs in all 3 areas (42%–61%), followed by RUTF costs, which is consistent with prior research.^5^^–^^7^^,^^17^^,^^31^^,^^32^ In the present study, the overall proportion of costs for CHW salaries was small at 1%–2% of the intervention costs. Yet, CHWs commented that sometimes, there are gaps in their payment. Given that their participation is essential to the success of this approach, future interventions of this type should ensure that sufficient funding is allocated to their salaries.

We found that institutional support, coordination, and supervision costs together accounted for a large proportion of overall costs, in this case, more than 50% in Kayes and Kita.

Our results suggest that additional supervision and support are needed to achieve acceptable health outcomes despite the high additional cost. Although the cost per child treated was significantly higher in Kayes and Kita, the cure rate in Bafoulabé was significantly lower than in the other 2 districts and did not meet Sphere Standards of at least 75% recovery.^15^ This is also reflected in the fact that while the cost per child treated in the base case was US$50–US$77 higher in the areas with additional supervision, the difference in cost per child cured in the base case estimate was lower at US$8–US$21 and uncertainty ranges overlapped. Interestingly, there was no discernible benefit of adding the nutrition-specific monthly supervision in Kita compared to Kayes. Charle et al. have previously suggested this lack of impact may indicate the need for better adaptation of the evaluation grids to the context and CHW sites.^15^ Furthermore, some of the items on the community-based management of acute nutrition checklist were already covered by iCCM supervision and may, therefore, not have led to additional impact on outcomes.

Results from our modeled sensitivity analysis indicate that the additional supervision has the potential to be a cost-effective strategy if efficiencies are made in its delivery. Replacing Action Against Hunger staff with government staff is an option. Other options include better integration of the nutrition-specific supervision into iCCM supervision activities and better adaptation of the said supervision to the CHW level, as also recommended by the technical committee of the project led by the Directorate of Nutrition of the Ministry of Health following the publication of the effectiveness results.^36^ Another alternative may be to start with high-intensity supervision when new CHWs are starting and then to reduce the supervision as they become more experienced.

This study had several strengths. It is one of few economic evaluations costing treatment of acute malnutrition integrated into the iCCM approach at scale. We conducted modeled scenario sensitivity analyses to understand the impact of scale and higher staff costs resulting from the involvement of NGO staff. The fact that it was not a randomized study but included all children in a relatively large area enabled us to cost the treatment more realistically outside of a trial context.

In line with our findings, since this study was conducted, the Malian government has put a new strategy of CHW supervision in place, which recommends employing and training 1 supervisor per 10–15 CHWs undertaking monthly supervision visits, where supervision is supported by a digital application which guides the workflow. The rollout of this strategy has started.

### Limitations

This study had several limitations. First, a number of assumptions/estimations had to be made regarding cost data that led to significant uncertainty around the cost estimates (as described in detail in the methods section), and data on RUTF consumption, length of stay, and baseline anthropometry were not available. It is possible that higher levels of supervision and technical support, which also included support to active case finding, in Kita and Kayes would have enabled earlier detection, which, in turn, could have led to quicker recovery, lower staff costs, and lower quantities of RUTF. The difference in cost per child treated and cured between Bafoulabé and 2 intervention areas, both in the base case and in the modeled equal scale scenario sensitivity analysis, may, therefore, be slightly overestimated. RUTF costs could also have been affected by different levels of defaulting and potentially more difficulties in assigning the correct weight-based dose in Bafoulabé, where CHWs had lower levels of education and less supportive supervision. Lack of availability of baseline anthropometric status and socioeconomic data for each participant also meant that the comparison of cure rates was not adjusted for baseline and other characteristics, which makes it difficult to completely disentangle the effect of the added supervision on effectiveness. Second, the treatment at CHWs was not rolled out simultaneously at all sites from the start of the project. It was not possible to obtain exact dates for all the sites. Therefore, the CHW-related costs, which were calculated based on similar start dates in the 3 districts, may have been slightly overestimated. However, the overall cost contribution of CHW salaries was small (less than 2%); therefore, more exact dates are unlikely to impact the overall result. Third, because of the different supervision levels in the districts, there was more quality control of the treatment outcome data in the intervention areas than in the control area. A further limitation is that we did not include inpatient treatment in total costs. Lastly, this study was not a randomized controlled trial, so the higher cure rates in Kita and Kayes cannot be solely attributed to the different levels of supervision and could have been influenced by other factors, such as the education level of CHWs, dietary factors, and prior knowledge of the approach in Kita.

## CONCLUSION

In conclusion, in this study, the additional supervision in Kita and Kayes significantly increased treatment costs. Although additional supervision came at a significant cost, results indicate that the additional supervision is needed to achieve satisfactory cure rates above Sphere Standards. Finally, the results of the sensitivity analysis indicate that the additional supervision has the potential to be a cost-effective strategy if supervision costs are reduced without compromising the quality of supervision, for example, by replacing INGO supervision staff with government staff. Further research should aim at better adapting the supervision model and associated tools to the context and investigate whether efficiencies can be made in its delivery.

## Supplementary Material

GHSP-D-23-00431_supplement.pdf

## Notes

**Peer Reviewed**

**First published online:** June 20, 2024
