# Supplementary material for: Integration of Acute Malnutrition Treatment Into Integrated Community Case Management in Three Districts in Southern Mali: An Economic Evaluation
Source: Glob Health Sci Pract. 2024 Jun 27;12(3):e2300431. doi: 10.9745/GHSP-D-23-00431 (PMC11216703; doi:10.9745/GHSP-D-23-00431)
Supplement: GHSP-D-23-00431_supplement.pdf [file GHSP-D-23-00431_supplement.pdf]

Supplement to: Cichon B, Lopez-Ejeda N, Mampindu MB, Bagayoko A, Samake M, Cuellar PC. Integration of acute malnutrition treatment into integrated community case management in three districts in southern Mali: an economic evaluation. *Glob Health Sci Pract*. 2024;12(3):e2300431. https://doi.org/10.9745/GHSP-D-23-00431

Supplementary Table 1. Community Health Worker supervision costs (base case)

**Activity and ingredients**	**Bafoulabé (Basic level of supervision)**	**Kayes (Medium level of supervision)**	**Kita (High level of supervision)**
Action Against Hunger staff	0	37822.3 (62.1%)	73968.3 (59%)
Department of Health and health centre staff	8829.2 (80.7%)	11917.1 (19.6%)	25755.3 (20.8%)
Transports, per diems, Accommodation, meeting space	2110.2 (19.3%)	11179.9 (18.4%)	24173.9 (19.5%)
**Total CHW supervision costs, US$ (%)**	10939.4 (100%)	60919.2 (100%)	123897.5 (100%)
Supervision cost per child treated at the CHW sites (base case)	34.95	174.05	235.10
Supervision cost per child recovered at the CHW sites (base case)	48.19	201.05	300.72
Supervision cost per child treated at the CHW sites (equal number of children treated per CHW site)[1]	53.6	225.6	235.10
Supervision cost per child recovered at the CHW sites (equal number of children treated per CHW site)[2]	73.9	260.6	300.72
Supervision cost per child treated at the CHW sites (larger scale scenario)[3]	9.3	27.1	27.5
Supervision cost per child recovered at the CHW sites (larger scale scenario)[3]	12.9	31.3	35.2

[1]In the base case, the average number of children treated per CHW site were 9 in Bafoulabé, 8 in Kayes and 6 in Kita. The CHW site supervision costs are dependent on the number of sites but not directly related to the number of children treated at each site. We therefore added a modelled estimate with the same number of children treated at each CHW site (i.e. 6 as in Kita) which means the total number of children treated in this analysis 204 in Bafoulabé, 270 in Kayes and 527 in Kita. [2]To calculate the number of children recovered in this analysis we applied the recovery rate of the study to the modelled number of children treated.[3]We also calculated a larger scale scenario where we assumed that 50 children would be treated at CHW sites in a year, this would equate to 90*50= 4500 in Kita, 45*50=2250 in Kayes and 34*50=1700 in Bafoulabé.

Supplement to: Cichon B, Lopez-Ejeda N, Mampindu MB, Bagayoko A, Samake M, Cuellar PC. Integration of acute malnutrition treatment into integrated community case management in three districts in southern Mali: an economic evaluation. *Glob Health Sci Pract*. 2024;12(3):e2300431. https://doi.org/10.9745/GHSP-D-23-00431

Supplemental table 2. Direct treatment cost per child treated and cured disaggregated by treatment site

	**Cost per child treated**	**Cost per child cured**
**Total programme**		
Bafoulabé	104.4 (80.7; 117.4)	155.9 (116.8; 182.8)
Kayes	95.2 (72.6; 107.1)	110.5 (83.3; 126.2)
Kita	98.3 (75.8; 110.5)	120.7 (92.0; 137.5)
**CHW site only**		
Bafoulabé	92.7 (69.9; 104.8)	127.8 (92.2; 153.7)
Kayes	96.7 (74.0; 108.7)	111.6 (83.1; 130.2)
Kita	148.3 (122.1; 164.2)	189.7 (151.4; 218.4)
**Health centre only**		
Bafoulabé	110.5 (86.3; 124.0)	173.3 (129.8; 205.8)
Kayes	94.9 (72.3; 106.7)	110.2 (82.9; 126.2)
Kita	88.8 (67.0; 100.3)	108.1 (80.6; 123.9)

Supplement to: Cichon B, Lopez-Ejeda N, Mampindu MB, Bagayoko A, Samake M, Cuellar PC. Integration of acute malnutrition treatment into integrated community case management in three districts in southern Mali: an economic evaluation. *Glob Health Sci Pract*. 2024;12(3):e2300431. https://doi.org/10.9745/GHSP-D-23-00431

Supplemental Table 3. Costs per child treated and cured in the base case and the modelled scenarios

	**Base Case**			**Model 1 (equal scale scenario) scenario[i]**			**Model 2 (Action Against supervision salary costs reduced to 1/3)[ii]**			**Model 3 (Equal scale and AAH salary costs reduced to 1/3)**		
	**Bafoulabé**	**Kayes**	**Kita**	**Bafoulabé**	**Kayes**	**Kita**	**Bafoulabé**	**Kayes**	**Kita**	**Bafoulabé**	**Kayes**	**Kita**
Total cost per child treated, US$ (95%CI)	203.4 (185.4; 222.7)	279.9 (246.9; 304.7)	253.9 (221.4; 274.8)	137.1 (113.4; 150.4)	245.2 (207.1; 258.1)	253.9 (221.4; 274.8)	203.4 (185.4; 222.7)	194.8 (168.4; 213.0)	190.0 (162.3; 205.9)	137.1 (113.4; 150.4)	173.5 (140.9; 180.8)	190.0 (162.3; 205.9)
Total cost per child recovered, US$ (95% CI)	303.9 (267.6; 346.4)	324.9 (282.9; 358.9)	311.8 (268.6; 342.0)	210.3 (170.9; 235.6)	297.5 (248.0; 317.7)	311.8 (268.6; 342.0)	303.9 (267.6; 346.4)	226.1 (193; 250.9)	233.3 (197; 256.2)	210.3 (170.9; 235.6)	210.4 (168.7; 222.6)	233.3 (197; 256.2)

[i]In the base case, the number of children treated at health centres and CHWs differed between the location. In the modelled equal scale scenario sensitivity analysis, the number of treatment sites, number of children as well the % of total number of children treated at the CHW sites were set to be equal and based on the numbers of children treated in Kita; [ii]In this scenario the numbers of children treated are as in the base case, but the costs of Action against Hunger supervision staff were reduced to 1/3 a number that was deemed to be feasible is staff were replaced by government staff.
